# Supplementary material for: Ecological and phylogenetic components of flatfish ectoparasites (Pleuronectiformes: Paralichthyidae) from the Southern Gulf of Mexico
Source: PLoS One. 2024 Oct 24;19(10):e0309818. doi: 10.1371/journal.pone.0309818 (PMC11500868; doi:10.1371/journal.pone.0309818)
Supplement: S1 Table — (DOCX) [file pone.0309818.s001.docx]

**S1 Table** Research cruises carried out in different regions along the continental shelf of the Southern Gulf of Mexico (SGoM).

| **Crusier** | **Region** | **Date** | **Sampling stations** | **Depths (m)** | **No. fish sampled** | **Sampling method** |
| --- | --- | --- | --- | --- | --- | --- |
| Gomex 1 | Yucatan Shelf | September 2010 | 8 | 10-130 | 25 | Shrimp trawls nets of 20 m long, with trawls of 50–60 min at 2.0–2.3 knots around each station |
| Gomex 2 |  | September 2011 | 19 | 15-200 | 58 |  |
| Gomex 3 |  | November 2012 | 13 | 40-200 | 24 |  |
| Gomex 4 |  | April 2016 | 18 |  | 161 |  |
| Gomex 5 |  | October 2017 | 15 |  | 99 |  |
| Gomex 6 |  | August 2018 | 14 |  | 109 |  |
| CO20015 | Tabasco-Campeche | October 2015 | 35 | 10-175 | 171 |  |
| KAB121-M1 |  | March 2008 | 17 | 12-35 | 202 |  |
| KAB121-M2 |  | July 2008 | 21 | 6-35 | 312 |  |
| LBA-AC02 | Veracruz- Tabasco | March 2018 | 9 | 100-300 | 45 | Shrimp trawls nets of 18 m long, with trawls of 10 min at 25 knots around each station |
| LBA-AC08 |  | March 2018 | 5 | 150-400 | 27 |  |
| LBA-AC18 |  | November 2018 | 3 | 4-12 | 15 |  |
| LBA-AC32 |  | November 2018 | 10 | 125-230 | 50 |  |
| LBA-050913 |  | December 2018 | 8 | 70-90 | 40 |  |
| Perdido 1 | Tamaulipas (Perdido) | May 2016 | 8 | 50-100 | 46 | Trawls with benthic sled on the seabed at a nautical speed mile. |
| Perdido 2 |  | Octoberr 2016 | 8 | 50-200 | 85 |  |
| Perdido 3 |  | June 2017 | 9 | 50-500 | 80 |  |
| Perdido 4 |  | October 2017 | 8 | 50-100 | 78 |  |
