# Supplementary material for: Ecological and phylogenetic components of flatfish ectoparasites (Pleuronectiformes: Paralichthyidae) from the Southern Gulf of Mexico
Source: PLoS One. 2024 Oct 24;19(10):e0309818. doi: 10.1371/journal.pone.0309818 (PMC11500868; doi:10.1371/journal.pone.0309818)
Supplement: S2 Table — N = total number of individuals collected. (DOCX) [file pone.0309818.s002.docx]

**S2 Table** Flatfish hosts species collected in the Southern Gulf of Mexico (SGoM) from the 18 oceanographic cruises (2010-2018) mentioned in **S1 Table**. N = total number of individuals collected.

| **Scientific name** | **Common name** | **N** | **Size range (cm)** | **Number of infested hosts** |
| --- | --- | --- | --- | --- |
| **Achiridae** | | | | |
| *Gymnachirus texae* Gunter | Gulf of Mexico fringed sole | 2 | 12.8-17 | 0 |
| *Trinectes maculatus* (Bloch & Schneider) | Hogchoker | 16 | 7.5-12.6 | 7 |
| **Bothidae** | | | | |
| *Bothus robinsi* Topp & Hoff | Twospot flounder | 15 | 14-24.2 | 0 |
| *Engyophrys senta* Ginsburg | American spiny flounder | 1 | 8.4 | 0 |
| *Monolene sessilicauda* Goode | Deepwater flounder | 10 | 8.5-14 | 0 |
| *Trichopsetta ventralis* (Goode & Bean) | Sash flounder | 171 | 6.4-35 | 4 |
| **Cynoglossidae** | | | | |
| *Symphurus piger* (Goode & Bean) | Deepwater tonguefish | 5 | 10.8-12.8 | 0 |
| **Paralichthyidae** | | | | |
| *Ancylopsetta dilecta* (Goode & Bean) | Three-eye flounder | 42 | 13.8-36.5 | 13 |
| *Ancylopsetta ommata* (Jordan & Gilbert) | Gulf of Mexico ocellated flounder | 1 | 30 | 0 |
| *Citharichthys cornutus* Günther | Horned whiff | 2 | 7-7.9 | 0 |
| *Citharichthys macrops* Dresel | Spotted whiff | 8 | 12.2-26.2 | 0 |
| *Citharichthys spilopterus* Günther | Bay whiff | 19 | 8.3-18.5 | 3 |
| *Cyclopsetta chittendeni* Bean | Mexican flounder | 695 | 7.5-39 | 31 |
| *Cyclopsetta fimbriata* (Goode & Bean) | Spotfin flounder | 23 | 19.7-38.6 | 1 |
| *Etropus crossotus* Jordan & Gilbert | Fringed flounder | 3 | 9.5-116 | 0 |
| *Gastropsetta frontalis* Bean | Shrimp flounder | 1 | 28.6-28.6 | 0 |
| *Syacium gunteri* Ginsburg | Shoal flounder | 146 | 6.6-38.3 | 10 |
| *Syacium micrurum* Ranzani | Channel flounder | 7 | 20-25 | 0 |
| *Syacium papillosum* (Linnaeus) | Dusky flounder | 450 | 8.5-36.6 | 108 |
| **Poecilopsettidae** | | | | |
| *Poecilopsetta beanii* Goode | Deepwater dab | 5 | 24.5-32.5 | 0 |
| Total | | 1622 |  | 177 |
