# Supplementary material for: Ecological and phylogenetic components of flatfish ectoparasites (Pleuronectiformes: Paralichthyidae) from the Southern Gulf of Mexico
Source: PLoS One. 2024 Oct 24;19(10):e0309818. doi: 10.1371/journal.pone.0309818 (PMC11500868; doi:10.1371/journal.pone.0309818)
Supplement: S3 Table — (DOCX) [file pone.0309818.s003.docx]

**S3 Table** ANOSIM analysis results for the sampling data**.**

|  | R Significance | |
| --- | --- | --- |
| Groups | Statistic | *P* value |
| 2008, 2016 | -0.333 | 1 |
| 2008, 2017 | 1 | 0.333 |
| 2008, 2018 | 0.583 | 0.220 |
| 2010, 2016 | -0.556 | 1 |
| 2010, 2017 | 0 | 0.667 |
| 2010, 2018 | -0.5 | 100 |
| 2011, 2016 | -0.556 | 100 |
| 2011, 2017 | 0 | 0.667 |
| 2011, 2018 | -0.333 | 0.80 |
| 2012, 2016 | -0.333 | 1 |
| 2012, 2017 | 0 | 0.667 |
| 2012, 2018 | -0.375 | 1 |
| 2015, 2016 | -0.556 | 1 |
| 2015, 2017 | 0 | 0.667 |
| 2015, 2018 | 0.417 | 0.40 |
| 2016, 2017 | -0.417 | 1 |
| 2016, 2018 | 0.009 | 0.429 |
| 2017, 2018 | -0.25 | 0.80 |
