# Supplementary material for: Ecological and phylogenetic components of flatfish ectoparasites (Pleuronectiformes: Paralichthyidae) from the Southern Gulf of Mexico
Source: PLoS One. 2024 Oct 24;19(10):e0309818. doi: 10.1371/journal.pone.0309818 (PMC11500868; doi:10.1371/journal.pone.0309818)
Supplement: Attachment — Submitted filename: comments_to_authors_R2.pdf [file pone.0309818.s008.pdf]

I commend the authors of the manuscript entitled “Ecological and phylogenetic components of flatfish ectoparasites” for their great work in improving clarity and coherence in this new version. My only suggestions are now regarding typographical errors, which are less frequent than in previous versions, but are still present. I list the ones I found below (referring to the revised manuscript without track-changes), but I would strongly recommend that a more careful proof-reading is done prior to publication.

L43. “evolutive history” should be “evolutionary history”.

L84-85. “from north-western end” should be “from the north-western end”

L169. “the Bray-Curtis” should be “a Bray-Curtis”

L195-198 Unless two ANOSIM analyses were performed, this is repeated with L183-184. I suggest L195-198 could be removed, and L183-184 adapted to provide any extra information needed. For example, L183-184 could be “Additionally, an analysis of similarities (ANOSIM) was performed for the sampling dates to check for potential effects on parasite communities of sampling across different years, and no significant year effect was found (Table S3).”

L204. Replace 'and of which' with 'all of which'

L215. There are two ‘host’ that should be plural. Replace ‘closely related host’ by ‘closely related hosts’, and ‘distant host’ by ‘distant hosts’

L261. “any the other factors” should be “any of the other factors”

L313-314. Should “was the sister species of species of the genus” be “was the sister species of the genus” or “was the sister species of a species of the genus”?

L362. Remove “the” from “that the nonspecific parasite species”

L432. “GoMS” should be “SGoM”.
