# Supplementary material for: Ecological and phylogenetic components of flatfish ectoparasites (Pleuronectiformes: Paralichthyidae) from the Southern Gulf of Mexico
Source: PLoS One. 2024 Oct 24;19(10):e0309818. doi: 10.1371/journal.pone.0309818 (PMC11500868; doi:10.1371/journal.pone.0309818)
Supplement: Attachment — Submitted filename: Comments-to-authors.docx [file pone.0309818.s004.docx]

In the manuscript entitled “Ecological and phylogenetic components of flatfish ectoparasites (Pleuronectiformes: Paralichthyidae) from the Southern Gulf of Mexico”, Soler-Jiménez et al. explore ectoparasite diversity patterns using an impressive number of flatfish recovered from many vessels that sampled over an extensive region in the Southern Gulf of Mexico. The authors attempt to answer whether phylogenetic congruence or ecological differences better explain flatfish ectoparasite diversity. The abstract is a good description of methods and results, but the broader context and its implications are missing. Because of that, the study questions and aims are not clear until the reader goes over the whole introduction. Overall, I find the study interesting and the questions relevant, however, there are many points for improvement. Firstly, there are confounding factors (host geographical structure, different sample sizes, generalist vs. specialist parasites, different sampling times and seasons) that need to be accounted for. There may be various ways to do so (e.g., rarefying), and I also suggest a few other analyses that could be undertaken and potentially further support the conclusions of the study. Among these analyses are investigations of distance-decay patterns, a separate phylogenetic congruence test for specialist and generalist parasites, and the use of a different genetic marker for fish phylogenies that is more appropriate for family-level comparisons (I noticed that the flatfish phylogenetic tree has many nodes of low bootstrap support, which could make phylogenetic congruence inferences even more challenging). In addition, a correction for multiple tests (at least) in pairwise comparisons must be made (or details on how it was done must be added). I also believe a more thorough description is warranted of how the different sample sizes affect analyses and the discovery of ectoparasites recovered from each flatfish species (ultimately affecting diversity measures). In terms of text structure, the results are repetitive with the information on tables and figures. Also, while the study system and the findings are the focus, including the broader context in the discussion would be beneficial. I left detailed comments below, split by major concern points and minor points. I believe that addressing such points will help towards having stronger evidence to explain the mechanisms underpinning the flatfish ectoparasite diversity patterns observed.

**Abstract**

The broader context is missing from the abstract, it goes straight into methods and results. No broader context or implications are mentioned at the end.

**L.12-13:** Write the species names in full the first time it is mentioned.

**Introduction**

**L.56**: “… during several oceanographic cruises”: Should seasonality be considered as a factor impacting parasite community composition? And if so, could the fact that flatfish were sampled in several cruises have impacted it? In other words, should there be a temporal component to the analyses? Is there an even representation of host and parasite species across time/seasons? If not, maybe a temporal analysis within places could be done, at least for Yucatan Shelf and Tabasco-Campeche, since both these regions have been sampled with the largest temporal range (around 7 years difference). Furthermore, comparisons among seasons could be carried out.

**Minor points - Introduction:**

**L.50:** Pleuronectiforms has capital P here, small p in line 46 - choose one for consistency and use throughout.

**L.50-51:** 127 species in the order Pleuronectiformes or 127 species of this order that occur in Mexico? Please clarify.

**Methods:**

**Table 1:** Some locations seem to have more sample stations than others. Is higher sampling effort correlated with the parasite community diversity within each of the regions? Also, various depths are included in all regions, but are sample sized similar for all depths? Different depths may have different host species, different ecology, and different parasites.

**L.123-125:** For the pairwise tests, a correction for multiple tests must be done (if this was done, please add details to methods).

**L.134-137:** Add more details for this. Did you include all host species or only the ones with non-zero parasites? What measures of richness were tested (mean abundance? prevalence? mean intensity?) Was this regression used to correct for sample size differences in other analyses? How?

**L.142:** cox1: Apologies if this is my misunderstanding, but you are building a tree to compare fish families, correct? Cox 1 is good at species level but evolves fast. 18S may be a more suitable marker for higher taxonomic ranks (such as family – see Tang et al., 2012 and Wu et al., 2015). Maybe you will have a better resolved tree with a different marker, and you can compare the results for COI and 18S too and understand at what level there is/isn't phylogenetic congruence in your data.

- Tang CQ, Leasi F, Obertegger U, Kieneke A, Barraclough TG, Fontaneto D, (2012). The widely used small subunit 18S rDNA molecule greatly underestimates true diversity in biodiversity surveys of the meiofauna. Proc Natl Acad Sci, 109(40):16208-12. doi: 10.1073/pnas.1209160109.
- Wu S, Xiong J, Yu Y, (2015). Taxonomic resolutions based on 18S rRNA genes: a case study of subclass copepoda. PLoS One, 10(6):e0131498. doi: 10.1371/journal.pone.0131498.

**L.145-146:** Were the sequences trimmed after aligning? Based on what criteria or at what length?

**L.152:** This is mostly looking at generalist parasites. Specialist parasites will be unique to fish species. Is the phylogenetic distance among parasites correlated with the phylogenetic distance among hosts? You can estimate the distances, build pairwise matrices and check for correlation between them (e.g., with a Mantel test). And you could do this for the specialist parasites only, and compare with the result when you include the generalists/generalists only analyses. Alternatively, there are methods such as ParaFit that will look at phylogenetic congruence using distances (Legendre et al., 2002, see also Hayward et al., 2021).

- Legendre P, Desdevises Y, Bazin E (2002). A statistical test for host-parasite coevolution. Syst Biol. 51(2):217-34. doi: 10.1080/10635150252899734.
- Hayward A, Poulin R, Nakagawa S, (2021). A broadscale analysis of hostsymbiont cophylogeny reveals the drivers of phylogenetic congruence. Ecology Letters. 24:1681–1696. <https://doi.org/10.1111/ele.13757>

**L.155:** “… were coded as 0 and 1…” - These analyses are considering only presence-absence of parasites. Could abundance be different between regions? Could that be included in the analysis?

**Minor points - Methods:**

**Figure 1:** The legend says 2010-2018, but Table 1 has a 2008 sampling date. I believe 2010 in the figure legend is wrong. If not, please clarify why 2008 is not included in the figure.

**Table 1:** some column names are not in English. Also, ‘Oceanographic vessel (OV) Justo Sierra’ is not a sampling method, it is the name of the vessel. Specify the method as done for the other regions.

**L.123:** ‘pair-wise’ – remove hyphen (pairwise)

**L.143:** So far you have only mentioned 22 species of fish, but not that they belong to different families. I suggest adding this information on line 79 (22 species belonging to 5? families).

**Results**

**L.165-166:** In the methods, you mentioned filtering for 5 parasite species (L. 127-128). Why here the sample size for fish with 'at least one ectoparasite species' is reported? Please report the sample size actually used in the analyses.

**L.166-167:** Based on Figure S1, there seems to be some geographical structure also in the hosts, with some fish species much more common in a region than in the others. This could have impacted the results, at least for specialist parasites. Was this considered somewhere?

**L.168:** “11 species” - This may be a low number for presence/absence analysis. Maybe abundance should be considered?

**L.171-172:** The different sample sizes for fish hosts is a little concerning. There are 450 *Citharichthys splilopterus*, so one would expect to find many parasite species for it, at least more than in fish species with sample sizes as small as the other three reported in line 172 (sample sizes ranging from 11-23). It would be interesting if comparisons involving absolute numbers (‘fish with largest numbers of parasites”) were made among fish of sample sizes with similar magnitude; relative values may be more informative to compare among fish with different sample sizes (such as the relative values on Table 3), but still, you expect to find more parasite species with larger sampling effort of host species. Could the different sample sizes be accounted for in the analyses? I know the authors report ‘no dependency relationship between the ectoparasite richness and the number of individuals of each host species’ in lines 173-175, but there needs to be more details on how this regression was done (see my comment for L134-137) and why it is enough to exclude potential effects of sampling size (see Gotelli et al., 2001 for a review and consider using a rarefaction analysis if the data allows).

- Gotelli, Nicholas J.; Colwell, Robert K. (2001). ["Quantifying biodiversity: procedures and pitfalls in the measurement and comparison of species richness"](https://doi.org/10.1046%2Fj.1461-0248.2001.00230.x). Ecology Letters. 4 (4): 379–391. [doi](https://en.wikipedia.org/wiki/Doi_(identifier)):[10.1046/j.1461-0248.2001.00230.x](https://doi.org/10.1046%2Fj.1461-0248.2001.00230.x).

**L.180-185:** This is repetitive with the information in Table 2.

**L.199:** Here again a species with large sample size and another with small sample size are compared (*C. chittendeni* with n=695 and *A. dilecta* with n = 37)

**L.204:** How are ‘important taxa’ defined? Is there a threshold, and if so, which one? Please clarify.

**L.217:** Why is p = 0.058 considered marginally significant, but p = 0.069 in L.175 considered non-significant? What is the alpha significance threshold for ‘marginally significant’?

**L.221-222:** These parasites have higher abundance compared with most of the others. Also, their collected fish hosts show some geographical structure (Figure S1), thus host species seems confounded with geography. For example, *A. dilecta* and *S. papillosum* were heavily sampled in Yucatan (both having *Cresseyus* sp1), but Tabasco-Campeche shows a more diverse sampling of fish hosts (according to Fig ureS1). The impact of these confounders, if not controlled for in the analyses, should at least be discussed.

**L.241-261:** the text is repetitive with Figure 4 (the trees). It is easier to see the described patterns in the figure than to understand a written description.

**Figure 4:** Note that many of the nodes have lower Bt support (below 80), and some are very low (Bt = 58), but how this could impact the results of phylogenetic congruence is not mentioned.

**Minor points – Results:**

**Table 3:** What are the 2 question marks in ‘Ancyrocephalidae gen. sp.??*’

**L.234:** ‘… 8 species’? I believe this is a typo. Were there not 10 parasitised fish species?

**L.241:** “congeneric species of the same genera’ – this is redundant. ‘congeneric’ means the ‘same genera’ - chose one or the other.

**Discussion**

**L.275-276:** ‘these variations seemed to be determined by a regional ecological component’ - There are some confounding factors with ecology, such as the distribution of the fish species sampled, as well as different seasons and different sample sizes for the fish hosts. It would be important to at least acknowledge these confounders in the discussion, as ‘regional ecological components’ do not seem to be the only alternative explanation to ‘phylogenetic congruence’ in this dataset.

**L.283-284:** ‘These similarities suggest that the phylogenetic affinity of the hosts is involved’ - Agreed, but these are three of the fish species with largest sample sizes, and generalist parasites. This is why I suggested splitting the analyses for generalists and specialists, as you may recover interesting patterns (which may or may not match).

**L.285-286:** ‘no evidence of congruent clustering’ - Have you tried to look at family level (or higher taxonomic ranks) than species level (such as I suggested with 18S in the methods?) The support for the ML tree is low for many of the clades, and I suspect this uncertainty in the ML tree is not helping to test for phylogenetic congruence.

**L.289-290:** What 'its own phylogenetic component' means? Please clarify.

**L.300-301:** The three sampled regions were subdivided based on geographical distance. What distance? was it an arbitrary cut-off? I wonder if including a distance-decay analysis (similarity of parasite communities and of fish hosts decreasing with geographical distance) would be more informative than using the three regions as categorical variables. Is there anything else different between these regions (habitat quality, environmental factors)? You describe known biogeographic barriers among these regions later in the discussion, and that these could prevent certain host species from migrating among these regions. Would that prevent parasites (and especially generalist parasites) as well?

**L.306-307:** One would have found a smaller number of the common parasites in fish species of smaller sample size. Were the different sample sizes accounted for in the analyses? How?

**L.316-317:** ‘Clearly, this group of species does not contribute to the similarity among the ectoparasite communities’ - Species-specific parasites may be more likely to have a phylogenetic signal than generalist parasites (see Hayward et al., 2021). Would it be possible to analyse species specific parasites separate from generalists? You could even compare both results, which would add another interesting point to the study.

- Hayward A, Poulin R, Nakagawa S, (2021). A broadscale analysis of hostsymbiont cophylogeny reveals the drivers of phylogenetic congruence. Ecology Letters. 24:1681–1696. <https://doi.org/10.1111/ele.13757>

**L.347-361:** How can the described biogeographical patterns influence fish hosts? Is there any study about the fish hosts distribution, genetic structure, etc? It may be relevant, in particular for specialist parasites, which will only occur where their fish host is available.

**Transition Discussion-Conclusion:** The discussion finishes abruptly, similar to the abstract. Overall, the text is very focused on the model system, and the broad context is mostly missing (it is partially mentioned in the conclusion, but since it is missing from the discussion, the conclusion feels like part of the discussion, as it has new information).

**Minor points - Discussion**

**L.347-353:** This is a single (very long) sentence. Please split it at least into two sentences for clarity.

**Conclusion**

**L.367-369:** The results of the analyses undertaken do not support ‘phylogenetic affinity’, so based on these results only, it could be ‘ruled out’. However, these analyses do not seem to harness the full potential of the data. There is more that could be done, such as splitting generalist from specialist parasites and accounting for the large differences in sample sizes.

**Minor points - Conclusion**

**L.370:** Do you mean ‘how many hosts are used’ by a parasite species? Please clarify.
