# Supplementary material for: Ecological and phylogenetic components of flatfish ectoparasites (Pleuronectiformes: Paralichthyidae) from the Southern Gulf of Mexico
Source: PLoS One. 2024 Oct 24;19(10):e0309818. doi: 10.1371/journal.pone.0309818 (PMC11500868; doi:10.1371/journal.pone.0309818)
Supplement: Attachment — Submitted filename: Response to Reviewers.docx [file pone.0309818.s005.docx]

Lilia C. Soler-Jiménez

Aquatic Pathology Laboratory

CINVESTAV

April 28, 2024

Professor Emily Chenette

Editor-in-Chief

Dear Dra. Emily Chenette

Attached you will find the manuscript entitled **“Ecological and phylogenetic components of flatfish ectoparasites (Pleuronectiformes: Paralichthyidae) from the Southern Gulf of Mexico”** by Lilia C. Soler-Jiménez, Frank A. Ocaña, David I. Hernández-Mena, Oscar A. Centeno-Chalé, Ma. Leopoldina Aguirre-Macedo and Víctor M. Vidal-Martínez. All the editorial suggestions were considered in this new version of the manuscript. Below you will find detailed answers to each one of these suggestions.

**AUTHOR(S):** Lilia C. Soler-Jiménez, Frank A. Ocaña, David I. Hernández-Mena, Oscar A. Centeno-Chalé, Ma. Leopoldina Aguirre-Macedo and Víctor M. Vidal-Martínez.

**Manuscript number:** PONE-D-23-31417

**TITLE:** Ecological and phylogenetic components of flatfish ectoparasites (Pleuronectiformes: Paralichthyidae) from the Southern Gulf of Mexico.

We are truly grateful with the referees for their valuable comments. We included first the referee´s suggestion and/or question, followed by our answer in a paragraph starting with A/, an acronym that stands for “Answer”, and in blue color.

Response to Reviewers

Additional Editor Comments:

Authors are invited to take note of the following in addition to the other comments
Lines 6-9- (Three regions……… the United States) not necessary in the abstract. Pleases delete.

A/ We appreciate the reviewer´s comment. We modified the abstract and omit this phrase.

Lines 12 & 13 write ‘’A. dilecta’’ and ‘’C. chittendeni’’ in full and subsequently use abbreviations for genus names. This should be reflected throughout the manuscript.

A/ We modified the name of each genre in full format in its first mention in the text and it was used as an abbreviation in its subsequent mentions (Line 17-18).

Table 1 column 7 provide the English version of the caption

A/ We modified the caption.

Line 79 Change – …’parasitologically examined’ to ‘’examined for ectoparasite infestation’’

A/ As suggested “parasitologically examined” was changed to “examined for ectoparasite infestation” (Line 128).

Line 82- Mention the method used for the identification, taxonomic keys or ??? and provide reference(s) for the method.

A/ The references for the taxonomic identification were included as suggested (Line 132-155).

Lines 92-93. I am not sure if this is ideal for metazoan ectoparasites- All specimens found were studied at 1,000 x magnification using immersion oil with an Olympus BX-50 light microscope (Olympus, Japan). Please modify

A/ The reviewer is right, for the study of metazoan ectoparasites not only 1000x magnification is used. The text was modified and corrected to avoid confusion (Line 139-141).

Line 142 italicize (cox 1)

A/ As suggested “cox 1” was changed to italics (Line 204).

Line 166- use infested instead of infected

A/ This line was removed.

Lines 164 -168- if total flatfish species were examined, and 10 were infested, then the negative shouldn’t be 13 species. Cross check please.

A/ We appreciate the reviewer's comment. This was a writing error, which has already been corrected in the text (Line 224-228).

Table 2- Add a row at the end of the table for the total of number of flatfish examined and the number infested

A/ We appreciate the reviewer's comment. A row at the end of the table for the total of number of flatfish examined and the number infested was added as suggested.

Table 3 caption- check spelling of ‘’different’’. Move ‘SD = standard deviation. N = total number of 193 individuals collected’ as footnotes under the table
A/ We appreciate the reviewer's comment. The suggested modifications were made.

Line 268- Correct -C) Parsimony ‘analysi’

A/ We appreciate the reviewer's comment. This was a writing error, which has already been corrected in the text (Line 335)

**Reviewer #1:**

The work is interesting, as it proposes to investigate a problem that is still widely discussed in the area of parasitological ecology. However, I made some observations that should be considered by authors so that the work is suitable for publication:

1 - the authors hypothesize that the structures of ectoparasite communities are the result of the geographic variability of the SGoM continental coast or whether the patterns depend on the phylogeny of the hosts (lines 57 to 62). However, no verification of the geographic hypothesis was carried out.

A/ We appreciate the reviewer´s comment. The geographic hypothesis was verified through the PERMANOVA statistical analysis, which considered a comparison of the ectoparasitic community structure between the different regions, showing statistically significant differences in the Yucatan Peninsula with respect to the other two regions.

2 - it is necessary for the authors to describe in more detail the region where the work was carried out, such as informing if there are seasonal changes in environmental characteristics (variables) such as water salinity, temperature, pH, productivity, ... , if there is a record of changes in the ichthyofauna over the years...

A/ We appreciate the reviewer's comment. The study area was described in more detail as suggested. A subsection was added to the materials and methods section with a detailed description of the area, where hydrological component were included.

3 - explain the reason for transforming the data, instead of using raw data. Explain why Euclidean distance is used as a dissimilarity/similarity metric, instead of, for example, the Bray-Curtis index.

A/ Data were transformed to reduce the contribution of highly abundant species in relation to less abundant ones as recommended by Anderson et al (2008). Indeed, the most appropriate distance method is the Bray-Curtis similarity, therefore, we have redone all analysis using this method.

4 - The authors present, as one of the results, a phylogeny where the families Bothidae and Paralichthydae both appear as non-monophyletic. I imagine that this phylogeny must result from the use of a single molecular marker, as well as the length of the sequence used. Recent studies demonstrate the monophyly of these families (see Campbell et al., 2019; Atta et al., 2022). Since the analyzes depend on a well-resolved phylogeny of the hosts, I suggest that the analyzes be redone with the organization of the phylogeny of the species considered in the study being appropriate to the best resolved phylogenies.

A/ We know in advance the disadvantages of carrying out phylogenetic analyzes with a single molecular marker, especially in fish, where genetic information is abundant to carry out phylogenies. However, the objective of our study is not to test the phylogenetic relationships and taxonomic classification of flatfishes. We only tried to show with a phylogeny that the fish that were parasitologically reviewed in this study are not closely related (except for *Syacium papillosum* and *Syacium gunteri*, which are sister species), to test whether the grouping of parasite communities is the result of a phylogenetic history or ecological processes.

Before choosing the genetic data set to perform the fish phylogeny, we first reviewed the specialized literature, including the studies that the reviewer cites in his commentary. We note that in general Paralichthyidae has been obtained as a non-monophyletic group: Campbell et al., 2019 divided it into Paralichthyidae I and II, but Paralichthyidae II is named as a new family that they called Cyclopsettidae; and Atta et al., 2022 also divided it into Paralichthyidae and Cyclopsettidae. The species that we include in our phylogeny belong to Paralichthyidae and Cyclopsettidae, that is, to Paralichthyidae I and II respectively. Since the taxonomy of flatfish continues to be modified by the addition of new genetic data from new or missing species, we decided to use the traditional classification for the moment. However, we have already modified it in the manuscript.

When we looked for genetic data that was common for all the fish species we analyzed in this study, we realized that, for example, exon capture data sets are not available for all species of interest. This also occurs with gene sequences, and the only gene that has been sequenced for all the flatfish in this study and that is available in public databases (such as Genbank) is cox 1. For us it was important to explore the phylogenetic position of all fish species without excluding any.

The use of a single gene, such as cox 1, to obtain phylogenies between organisms from different families can has several problems in the phylogenetic result and in obtaining low clade support values. However, the phylogenetic tree of cox1 in general had a topology similar to the trees generated with more genetic data and with a greater number of taxa.

In summary, we decided to only use sequences from cox 1 due to: 1) the availability of sequences for all fish species analyzed in this study; 2) the phylogenetic tree obtained with cox 1 is similar to others that have been obtained with larger data sets. 3) the objective of the analysis was only to demonstrate that the reviewed flatfish do not have a close phylogenetic relationship.

**Minor reviews:**

Line 23 - change to "the ecology and phylogeny of hosts as directors";

A/ As suggested “drivers” was changed to “directors”.

Line 25 - change to "host species is a better predictor";

A/ As suggested the word “host” was included in the phase (line 64).

Lines 62 to 64 - this prediction is valid if the phylogeny of the hosts is the main or only factor that directs the patterns of ectoparasite community structures. However, another hypothesis was also suggested, so the prediction of this hypothesis must also be presented;

A/ The hypotheses and objectives were modified as suggested (lines 120-127).

Lines 64 to 66 - the objectives are to test the hypotheses that have been suggested;

A/ The hypotheses and objectives were modified as suggested (lines 120-127).

Lines 69, 70 - inform the duration of each "Research cruises"

A/ The information for each cruise was detailed in Table S1 of the Supporting information.

Table 1 - the name of the last column is in Spanish, translate into English; tables do not contain the internal drawn lines;

A/ We modified the caption and tables format.

Lines 87, 88 - Insert Vidal-Martínez and colleagues before reference [26];

A/ We modify the text as suggested.

Line 96 - replace "&" with "and";

A/ We modify the text as suggested.

Line 98 - replace "et al." by "colleagues" before reference [28];

A/ We modify the text as suggested.

Line 103 - Insert Bush et al. before reference [31];

A/ We modify the text as suggested.

Line 117, 118 - the term parasite infracommunity was defined in lines 109 and 110, therefore, the authors should remove or use this definition in lines 109, 110;

A/ The reviewer is correct. We remove the definition from lines 117 and 118.

Line 164 - this information has already been presented on line 79;

A/ The reviewer is correct. The information is only repeated to introduce and give references to the reader.

Table 2 - tables do not contain the internal drawn lines;

A/ We modified the tables format.

Table 3 - tables do not contain the internal drawn lines;

A/ We modified tables format.

Line 324 - replace "shelf" with "Shelf" ;

A/ We homogenize the writing of the “Yucatan Shelf” throughout the text, with a capital S.

Line 386 - correct the name of the periodical;

A/ We modified as suggested.

**Reviewer #2:**

In the manuscript entitled “Ecological and phylogenetic components of flatfish ectoparasites (Pleuronectiformes: Paralichthyidae) from the Southern Gulf of Mexico”, Soler-Jiménez et al. explore ectoparasite diversity patterns using an impressive number of flatfish recovered from many vessels that sampled over an extensive region in the Southern Gulf of Mexico. The authors attempt to answer whether phylogenetic congruence or ecological differences better explain flatfish ectoparasite diversity.

The abstract is a good description of methods and results, but the broader context and its implications are missing. Because of that, the study questions and aims are not clear until the reader goes over the whole introduction.

A/ We appreciate the reviewer´s comment. The reviewer is right. Therefore, the abstract has already been modified, a paragraph was included where the reader is put in context for a better understanding of it:

“For many years, the relative importance of phylogeny and ecology as drivers of parasite community structure has been a topic of debate among parasite ecologists. Here, we address this issue using data on the metazoan ectoparasite communities of different flatfish species.”

Overall, I find the study interesting and the questions relevant, however, there are many points for improvement. Firstly, there are confounding factors (host geographical structure, different sample sizes, generalist vs. specialist parasites, different sampling times and seasons) that need to be accounted for. There may be various ways to do so (e.g., rarefying), and I also suggest a few other analyses that could be undertaken and potentially further support the conclusions of the study. Among these analyses are investigations of distance-decay patterns, a separate phylogenetic congruence test for specialist and generalist parasites, and the use of a different genetic marker for fish phylogenies that is more appropriate for family-level comparisons (I noticed that the flatfish phylogenetic tree has many nodes of low bootstrap support, which could make phylogenetic congruence inferences even more challenging).

A/ Detailed responses to these observations can be found below.

In addition, a correction for multiple tests (at least) in pairwise comparisons must be made (or details on how it was done must be added). I also believe a more thorough description is warranted of how the different sample sizes affect analyses and the discovery of ectoparasites recovered from each flatfish species (ultimately affecting diversity measures).

A/ The pairwise test used was the t-test that uses permutations. We advocate for the explanation given by Anderson et al. (2008).

***“Unlike traditional statistics packages, P-values for all pair-wise tests (t-test) in PERMANOVA are obtained using permutations, not tables…***

***… The permutation P-values do provide an exact test of each individual null hypothesis of interest. In contrast, most ad hoc experiment-wise corrections that could be used here (such as Bonferroni) are inexact and known to be overly conservative…”***

On the other hand, PERMANOVA is a permutation-based technique that makes no distributional assumptions about multivariate normality or homogeneity of variances. This analysis is based on permutation tests that can be used for unbalanced designs with reasonable sample sizes. In our case, we considered n=5 as a minimum reasonable sample size.

In terms of text structure, the results are repetitive with the information on tables and figures.

A/ The structure of the text was changed as suggested, so as not to repeat information at different points in the manuscript.

Also, while the study system and the findings are the focus, including the broader context in the discussion would be beneficial.

A/ The discussion was changed as suggested, considering a broader framework of different points of view and including more general factors influencing the structure of ectoparasitic flatfish communities.

**Abstract**

The broader context is missing from the abstract, it goes straight into methods and results. No broader context or implications are mentioned at the end.

A/ The reviewer is right. Therefore, the abstract has already been modified, a paragraph was included where the reader is put in context for a better understanding of it:

“For many years, the relative importance of phylogeny and ecology as drivers of parasite community structure has been a topic of debate among parasite ecologists. Here, we address this issue using data on the metazoan ectoparasite communities of different flatfish species.”

**L.12-13:** Write the species names in full the first time it is mentioned.

A/ We modified the name of each species in full format in its first mention in the text and it was used as an abbreviation in its subsequent mentions.

**Introduction**

**L.56**: “… during several oceanographic cruises”: Should seasonality be considered as a factor impacting parasite community composition? And if so, could the fact that flatfish were sampled in several cruises have impacted it? In other words, should there be a temporal component to the analyses? Is there an even representation of host and parasite species across time/seasons? If not, maybe a temporal analysis within places could be done, at least for Yucatan Shelf and Tabasco-Campeche, since both these regions have been sampled with the largest temporal range (around 7 years difference). Furthermore, comparisons among seasons could be carried out.

A/ As suggested, seasonality was included in the analyzes because it is considered an important factor in the structure of the ectoparasitic community. For this, we additionally performed an analysis of similarities (ANOSIM) for the sampling data. However, no relevant year effect was found that affects the structure of the ectoparasitic community of the different species of flatfish collected in different cruises (see the following table).

**Table** ANOSIM analysis results for the sampling dates**.**

|  | R Significance | |
| --- | --- | --- |
| Groups | Statistic | *P* value |
| 2008, 2016 | -0.333 | 1 |
| 2008, 2017 | 1 | 0.333 |
| 2008, 2018 | 0.583 | 0.220 |
| 2010, 2016 | -0.556 | 1 |
| 2010, 2017 | 0 | 0.667 |
| 2010, 2018 | -0.5 | 100 |
| 2011, 2016 | -0.556 | 100 |
| 2011, 2017 | 0 | 0.667 |
| 2011, 2018 | -0.333 | 0.80 |
| 2012, 2016 | -0.333 | 1 |
| 2012, 2017 | 0 | 0.667 |
| 2012, 2018 | -0.375 | 1 |
| 2015, 2016 | -0.556 | 1 |
| 2015, 2017 | 0 | 0.667 |
| 2015, 2018 | 0.417 | 0.40 |
| 2016, 2017 | -0.417 | 1 |
| 2016, 2018 | 0.009 | 0.429 |
| 2017, 2018 | -0.25 | 0.80 |

**Minor points - Introduction:**

**L.50:** Pleuronectiforms has capital P here, small p in line 46 - choose one for consistency and use throughout.

A/ We homogenize the writing of the word “Pleuronectiforms” throughout the text, with a capital P.

**L.50-51:** 127 species in the order Pleuronectiformes or 127 species of this order that occur in Mexico? Please clarify.

A/ There are 127 species of this order found in Mexico. This aspect was clarified in the text:

“In Mexico, Pleuronectiforms are an abundant group of fish (with approximately 127 species of this order), some of them with high commercial value [19]”.

**Methods:**

**Table 1:** Some locations seem to have more sample stations than others. Is higher sampling effort correlated with the parasite community diversity within each of the regions? Also, various depths are included in all regions, but are sample sized similar for all depths? Different depths may have different host species, different ecology, and different parasites.

A/ We appreciate the reviewer's comment. As the reviewer indicates, some of the localities have more sampling stations than others, so at first glance it could be inferred that the sampling effort is correlated with the diversity of the parasite community in each of the regions. However, in the diversity analysis using PERMANOVA different sample sizes can be handled by using random permutations. Instead of making direct comparisons between samples with different sizes, random permutations are generated to redistribute the data and create sample groups with similar sizes. This ensures that comparisons are made fairly and that variability in sample sizes is accounted for in the analysis. Additionally, an ANOVA was also performed between the diversity of parasite species for each region (which was not included in the study), showing us that the differences in the median values among the treatment groups are not great enough to exclude the possibility that the difference is due to random sampling variability; Therefore, there is not a statistically significant difference among diversity per regions (P = 0.238).

Regarding depth, this component was considered as a covariate in the new analyses, due to the importance in the parasite composition of flounder species (Alarcos and Timi, 2012; Oliva et al., 2004). We found a significant potential effect of depth in parasite composition. However, this effect seemed to be constant among regions and hosts since there were not significant interactions with the other factors.

**L.123-125:** For the pairwise tests, a correction for multiple tests must be done (if this was done, please add details to methods).

A/ We appreciate the reviewer's comment. The pairwise test used was the t-test that uses permutations. We advocate for the explanation given by Anderson et al. (2008).

***“Unlike traditional statistics packages, P-values for all pair-wise tests (t-test) in PERMANOVA are obtained using permutations, not tables…***

***… The permutation P-values do provide an exact test of each individual null hypothesis of interest. In contrast, most ad hoc experiment-wise corrections that could be used here (such as Bonferroni) are inexact and known to be overly conservative…”***

**L.134-137:** Add more details for this. Did you include all host species or only the ones with non-zero parasites? What measures of richness were tested (mean abundance? prevalence? mean intensity?) Was this regression used to correct for sample size differences in other analyses? How?

A/ The criteria for including hosts in the statistical analyzes were: parasitized host species present in all regions and with more than 5 individuals in each region.

Qualitative attributes used to characterize flatfish ectoparasitic communities were abundance, mean abundance, prevalence and mean intensity according to (Bush et al. 1997). Values described for each parasite found in table 1 of the manuscript.

Regarding the regression analysis, this was carried out to test the independence of ectoparasite richness with respect to the number of individuals per host species. However, we decided to eliminate it because it was not really necessary. PERMANOVA is a permutation-based technique that makes no distributional assumptions about multivariate normality or homogeneity of variances. This analysis is based on permutation tests that can be used for unbalanced designs with reasonable sample sizes. Instead of making direct comparisons between samples with different sizes, random permutations are generated to redistribute the data and create sample groups with similar sizes. This ensures that comparisons are made fairly and that variability in sample sizes is accounted for in the analysis.

**L.142:** cox1: Apologies if this is my misunderstanding, but you are building a tree to compare fish families, correct? Cox 1 is good at species level but evolves fast. 18S may be a more suitable marker for higher taxonomic ranks (such as family – see Tang et al., 2012 and Wu et al., 2015). Maybe you will have a better resolved tree with a different marker, and you can compare the results for COI and 18S too and understand at what level there is/isn't phylogenetic congruence in your data.

- Tang CQ, Leasi F, Obertegger U, Kieneke A, Barraclough TG, Fontaneto D, (2012). The widely used small subunit 18S rDNA molecule greatly underestimates true diversity in biodiversity surveys of the meiofauna. Proc Natl Acad Sci, 109(40):16208-12. doi: 10.1073/pnas.1209160109.
- Wu S, Xiong J, Yu Y, (2015). Taxonomic resolutions based on 18S rRNA genes: a case study of subclass copepoda. PLoS One, 10(6):e0131498. doi: 10.1371/journal.pone.0131498.

A/ Indeed we are constructing a phylogenetic tree to investigate the phylogenetic relationships of the host species reviewed, but it is also to demonstrate that these hosts do not have a close phylogenetic relationship (except for *Syacium papillosum* and *Syacium gunteri*, which are sister species). We agree that cox 1 has limits at the family level due to its substitution rate and that there are more informative genes at that level. However, for us it was important that all fish species reviewed in this study were represented in the phylogeny, and the only gene that has been sequenced for all of them is cox 1.

**L.145-146:** Were the sequences trimmed after aligning? Based on what criteria or at what length?

A/ The sequences were not trimmed, they were used as they were published in Genbank.

**L.152:** This is mostly looking at generalist parasites. Specialist parasites will be unique to fish species. Is the phylogenetic distance among parasites correlated with the phylogenetic distance among hosts? You can estimate the distances, build pairwise matrices and check for correlation between them (e.g., with a Mantel test). And you could do this for the specialist parasites only, and compare with the result when you include the generalists/generalists only analyses. Alternatively, there are methods such as ParaFit that will look at phylogenetic congruence using distances (Legendre et al., 2002, see also Hayward et al., 2021).

- Legendre P, Desdevises Y, Bazin E (2002). A statistical test for host-parasite coevolution. Syst Biol. 51(2):217-34. doi: 10.1080/10635150252899734.
- Hayward A, Poulin R, Nakagawa S, (2021). A broadscale analysis of hostsymbiont cophylogeny reveals the drivers of phylogenetic congruence. Ecology Letters. 24:1681–1696. <https://doi.org/10.1111/ele.13757>

A/ Most parasites are generalists and are from different groups of invertebrates. Unfortunately for most of the parasite species in this study (both generalists and specialists) we do not have genetic data that would allow us to make phylogenetic inferences. Therefore, a coevolutionary comparison or estimation of parasite/host phylogenetic distances is complicated. We also want to comment that the objective of our analyses was to investigate whether or not the assembly of parasite communities has a relationship with the phylogeny of the hosts, rather than the coevolution between parasites and specific hosts.

**L.155:** “… were coded as 0 and 1…” - These analyses are considering only presence-absence of parasites. Could abundance be different between regions? Could that be included in the analysis?

A/ Abundance is an ecological and not an evolutionary parameter. As the objective of this analysis was to relate parasites to their hosts to see if the affinities between them have any evolutionary explanation, the abundance parameter takes a backseat.

**Minor points - Methods:**

**Figure 1:** The legend says 2010-2018, but Table 1 has a 2008 sampling date. I believe 2010 in the figure legend is wrong. If not, please clarify why 2008 is not included in the figure.

A/ Thank you for your comment, there is indeed an error in the legend of the figure which has already been corrected.

**Table 1:** some column names are not in English. Also, ‘Oceanographic vessel (OV) Justo Sierra’ is not a sampling method, it is the name of the vessel. Specify the method as done for the other regions.

A/ The caption in the Table 1 was modified. Likewise, the name of the vessel was removed and the sampling method was specified.

**L.123:** ‘pair-wise’ – remove hyphen (pairwise)

A/ The hyphen was removed as suggested.

**L.143:** So far you have only mentioned 22 species of fish, but not that they belong to different families. I suggest adding this information on line 79 (22 species belonging to 5? families).

A/ Family information was included as suggested: “Twenty-two flatfish species belonging to five families were collected”

**Results**

**L.165-166:** In the methods, you mentioned filtering for 5 parasite species (L. 127-128). Why here the sample size for fish with 'at least one ectoparasite species' is reported? Please report the sample size actually used in the analyses.

A/ We want to clarify that in this part we made a general description of the parasitized hosts. The sample sizes used and the inclusion criteria of the hosts for the statistical analyzes are described in lines 173 and 174.

**L.166-167:** Based on Figure S1, there seems to be some geographical structure also in the hosts, with some fish species much more common in a region than in the others. This could have impacted the results, at least for specialist parasites. Was this considered somewhere?

A/ Yes, this point was considered in the discussion section (Line 392-399).

“Regarding the conformation of the ectoparasite communities throughout the study area, the results showed that the Yucatan shelf region presents differences with respect to Tabasco-Campeche and Tamaulipas-Veracruz. In this case, the variability in the ectoparasite community detected among regions could be explained by (1) the host composition per region, which can be determined by landscape topography, which in turn, may constrain either direct or host-mediated dispersal of ectoparasite species. The similarities in the ectoparasite community structure in the Tamaulipas-Veracruz and Tabasco-Campeche regions, and at the same time, their differentiation with respect to the Yucatan Shelf, can be attributed to the composition of parasitized hosts in each region. While Tamaulipas-Veracruz and Tabasco-Campeche presented a very similar host species composition (both in relative abundance and in species richness), the set of host species of the Yucatan Shelf is composed mostly of individuals of the species *S. papillosum*”.

**L.168:** “11 species” - This may be a low number for presence/absence analysis. Maybe abundance should be considered?

A/ Thank you very much for your comment. For all statistical analyzes the relative abundances of the ectoparasites were used. The concept of presence/absence was used in the phylogenetic analysis, since abundance is an ecological and not an evolutionary parameter. As the objective of this analysis was to relate parasites to their hosts to see if the affinities between them have any evolutionary explanation, the abundance parameter takes a backseat.

**L.171-172:** The different sample sizes for fish hosts is a little concerning. There are 450 *Citharichthys splilopterus*, so one would expect to find many parasite species for it, at least more than in fish species with sample sizes as small as the other three reported in line 172 (sample sizes ranging from 11-23). It would be interesting if comparisons involving absolute numbers (‘fish with largest numbers of parasites”) were made among fish of sample sizes with similar magnitude; relative values may be more informative to compare among fish with different sample sizes (such as the relative values on Table 3), but still, you expect to find more parasite species with larger sampling effort of host species. Could the different sample sizes be accounted for in the analyses? I know the authors report ‘no dependency relationship between the ectoparasite richness and the number of individuals of each host species’ in lines 173-175, but there needs to be more details on how this regression was done (see my comment for L134-137) and why it is enough to exclude potential effects of sampling size (see Gotelli et al., 2001 for a review and consider using a rarefaction analysis if the data allows).

- Gotelli, Nicholas J.; Colwell, Robert K. (2001). ["Quantifying biodiversity: procedures and pitfalls in the measurement and comparison of species richness"](https://doi.org/10.1046%2Fj.1461-0248.2001.00230.x). Ecology Letters. 4 (4): 379–391. [doi](https://en.wikipedia.org/wiki/Doi_(identifier)):[10.1046/j.1461-0248.2001.00230.x](https://doi.org/10.1046%2Fj.1461-0248.2001.00230.x).

A/ The reviewer is correct and we understand your concern. Therefore, for this revised version, a rarefaction analysis for ectoparasite diversity in the parasitized host species was carried out, which was included in the manuscript. The rarefaction analysis indicated that the only host species that reach asymptote was *S. papillosum*. Therefore, the analyzes were based on observed ectoparasite communities, but not be representative of the “true” ectoparasite diversity from flatfish.

Regarding differences in sample sizes by host included in the statistical analysis, these are resolved in the PERMANOVA diversity analysis using random permutations. In this analysis, instead of making direct comparisons between samples of different sizes, random permutations are generated to redistribute the data and create groups of samples with similar sizes. This ensures that comparisons are made fairly and that variability in sample sizes is considered in the analysis (Anderson et al. 2008).

**L.180-185:** This is repetitive with the information in Table 2.

A/ The information on these lines was changed as suggested, so that it is not repeated with the information in table 1.

**L.199:** Here again a species with large sample size and another with small sample size are compared (*C. chittendeni* with n=695 and *A. dilecta* with n = 37)

A/ We appreciate the reviewer's comment. Indeed, in the analysis the structural ectoparasitic communities is compared between four hosts with different sample sizes (*C. chittendeni* with n=695, *A. dilecta* with n = 37, *S. papillosum* with n=450 and *S. gunteri* with n=146). However, in a diversity analysis using PERMANOVA different sample sizes can be handled by using random permutations. Instead of making direct comparisons between samples with different sizes, random permutations are generated to redistribute the data and create sample groups with similar sizes. This ensures that comparisons are made fairly and that variability in sample sizes is accounted for in the analysis. On the other hand, I consider that n=37 is not a small sample size.

**L.204:** How are ‘important taxa’ defined? Is there a threshold, and if so, which one? Please clarify.

A/ In the SIMPER analysis, the "important taxa" refer to the species that contribute significantly to the observed differences between hosts, that is, they are the species that have the greatest impact on the observed differences in the structure of ectoparasite communities between hosts.

**L.217:** Why is p = 0.058 considered marginally significant, but p = 0.069 in L.175 considered non-significant? What is the alpha significance threshold for ‘marginally significant’?

A/ We appreciate the reviewer's comment. This was a drafting error. The texts were modified and this sentence was removed. Note that statistical analyzes were redone.

**L.221-222:** These parasites have higher abundance compared with most of the others. Also, their collected fish hosts show some geographical structure (Figure S1), thus host species seems confounded with geography. For example, *A. dilecta* and *S. papillosum* were heavily sampled in Yucatan (both having *Cresseyus* sp1), but Tabasco-Campeche shows a more diverse sampling of fish hosts (according to Fig ureS1). The impact of these confounders, if not controlled for in the analyses, should at least be discussed.

A/ The reviewer is right. Therefore, this point was considered in the discussion section (Line 392-399).

“Regarding the conformation of the ectoparasite communities throughout the study area, the results showed that the Yucatan shelf region presents differences with respect to Tabasco-Campeche and Tamaulipas-Veracruz. In this case, the variability in the ectoparasite community detected among regions could be explained by (1) the host composition per region, which can be determined by landscape topography, which in turn, may constrain either direct or host-mediated dispersal of ectoparasite species. The similarities in the ectoparasite community structure in the Tamaulipas-Veracruz and Tabasco-Campeche regions, and at the same time, their differentiation with respect to the Yucatan Shelf, can be attributed to the composition of parasitized hosts in each region. While Tamaulipas-Veracruz and Tabasco-Campeche presented a very similar host species composition (both in relative abundance and in species richness), the set of host species of the Yucatan Shelf is composed mostly of individuals of the species *S. papillosum*”.

**L.241-261:** the text is repetitive with Figure 4 (the trees). It is easier to see the described patterns in the figure than to understand a written description.

A/ Although the figures and the text can give the same information, we think it is convenient to highlight in the text the most important results that were observed in the trees and that have to do directly with the host species that we reviewed in this study.

**Figure 4:** Note that many of the nodes have lower Bt support (below 80), and some are very low (Bt = 58), but how this could impact the results of phylogenetic congruence is not mentioned.

A/ We think that the low support values for the clades can be interpreted as a weakness of the analysis. However, this is relevant if the host species that were parasitologically reviewed were more closely phylogenetically related. But most of the fish in this study belong to different families and genera. The exception is *Cyclopsetta* and *Syacium* species, but these clades have higher support values. Furthermore, regarding the relationships of families and genera, the phylogeny obtained in this study has a topology similar to that obtained with a larger data set (see Campbell et al., 2019).

**Minor points – Results:**

**Table 3:** What are the 2 question marks in ‘Ancyrocephalidae gen. sp.??*’

A/ It was a typo, this has already been corrected, as you suggested.

**L.234:** ‘… 8 species’? I believe this is a typo. Were there not 10 parasitised fish species?

A/ The alignment matrix, in the phylogenetic analysis, was built with sequences of 8 species (*Ancylopsetta* sp. and *Syacium* sp. were not included).

**L.241:** “congeneric species of the same genera’ – this is redundant. ‘congeneric’ means the ‘same genera’ - chose one or the other.

A/ It was a typo, this has already been corrected, as you suggested.

“In particular, the parasitized species in this study formed monophyletic groups with their respective congeners”

**Discussion**

**L.275-276:** ‘these variations seemed to be determined by a regional ecological component’ - There are some confounding factors with ecology, such as the distribution of the fish species sampled, as well as different seasons and different sample sizes for the fish hosts. It would be important to at least acknowledge these confounders in the discussion, as ‘regional ecological components’ do not seem to be the only alternative explanation to ‘phylogenetic congruence’ in this dataset.

A/ The reviewer is correct in his comment, it is for this reason that in the new statistical analyzes, ecological factors such as the depth and size of the hosts were considered as covariates, because it has been reported that these two variables affect the parasite composition of the flounder species (Alarcos and Timi, 2012; Oliva et al., 2004).

Regarding differences in sample sizes by host and number of stations, these are resolved in the PERMANOVA diversity analysis using random permutations. In this analysis, instead of making direct comparisons between samples of different sizes, random permutations are generated to redistribute the data and create groups of samples with similar sizes. This ensures that comparisons are made fairly and that variability in sample sizes is considered in the analysis (Anderson et al. 2008). Likewise, factors such as the distribution of host species were analyzed in the discussion section.

**L.283-284:** ‘These similarities suggest that the phylogenetic affinity of the hosts is involved’ - Agreed, but these are three of the fish species with largest sample sizes, and generalist parasites. This is why I suggested splitting the analyses for generalists and specialists, as you may recover interesting patterns (which may or may not match).

A/ We agree with the reviewer, according to the study recently carried out by Osuna-Cabanillas et al 2024, when analyzing specialist and generalist parasites separately, different behaviors are elucidated than in a joint analysis. However, in this study, when analyzing only non-specific parasite species, differences were detected between all host combinations except between *S. gunteri* and *S. papillosum*. For the analysis with only the parasitic species specific to their host, no differences were detected between the hosts, this is perhaps because the sample sizes are too small, and this may cause little ability to detect significant differences between the groups. On the other hand, in both cases significant differences are detected for the region, making it very clear that the Yucatan Peninsula is different from the other two regions.

**L.285-286:** ‘no evidence of congruent clustering’ - Have you tried to look at family level (or higher taxonomic ranks) than species level (such as I suggested with 18S in the methods?) The support for the ML tree is low for many of the clades, and I suspect this uncertainty in the ML tree is not helping to test for phylogenetic congruence.

A/ We appreciate the reviewer´s comment. However, our interest was to build a phylogenetic tree to investigate phylogenetic relationships at the level of host species and demonstrate that these hosts do not have a close phylogenetic relationship. Likewise, it is important for us that all fish species reviewed were represented in the phylogeny, and the only gene that has been sequenced for all of them is cox 1.

**L.289-290:** What 'its own phylogenetic component' means? Please clarify.

A/ This phrase was removed to avoid confusion. Also, the introduction section was modified to clarify the meaning of the ecological and phylogenetic components of the parasite fauna of the flatfish species studied.

**L.300-301:** The three sampled regions were subdivided based on geographical distance. What distance? was it an arbitrary cut-off? I wonder if including a distance-decay analysis (similarity of parasite communities and of fish hosts decreasing with geographical distance) would be more informative than using the three regions as categorical variables. Is there anything else different between these regions (habitat quality, environmental factors)? You describe known biogeographic barriers among these regions later in the discussion, and that these could prevent certain host species from migrating among these regions. Would that prevent parasites (and especially generalist parasites) as well?

A/ This study was carried out from the coast of Tamaulipas to the Yucatán Peninsula, considering three geographic regions according to work previously carried out by Salmerón-García et al. [38] and Quintanilla-Mena et al [2020], which determine this regionalization due to differences in meteorological and physical oceanographic processes, river flows and topographic characteristics. All samples were obtained during oceanographic cruises carried out for the hydrocarbon industry in this region. In that sense, all this information was obtained from opportunity sampling. Consequently, the information comes from non-probabilistic sampling. In this sense, we have limitations to analyze the data properly. However, we consider it important to publish these data since it is extremely expensive to obtain all this parasitological information even when we have limitations in data analysis. However, distance-decay analysis was performed to test the decay of ectoparasite community similarity over distance for all hosts and was included as suggested. The analysis found no decay pattern of ectoparasite similarity over distance in any of the four flatfish hosts (*S. gunteri*: Rho = -0.06, P = 0.733; *S. papillosum*: Rho = 0.07, P = 0.203; *C. chittendeni*: Rho = 0.02, P = 0.387; *A. dilecta*: Rho = 0.29, P = 0.142).

**L.306-307:** One would have found a smaller number of the common parasites in fish species of smaller sample size. Were the different sample sizes accounted for in the analyses? How?

A/ It is true that the sample size for each host species is different. This is because all this information was obtained from opportunity sampling. Consequently, the information comes from non-probabilistic sampling. In this sense, we have limitations to analyze the data properly. Initially, a rarefaction analysis was carried out for the diversity of ectoparasites in the parasitized host species, and as expected, only the host species with the largest sample size reached asymptote (i.e., *S. papillosum*). To remedy this, a diversity analysis was carried out using PERMANOVA, in which different sample sizes can be handled by using random permutations. Instead of making direct comparisons between samples of different sizes, random permutations are generated to redistribute the data and create groups of samples with similar sizes. This ensures that comparisons are made fairly and that variability in sample sizes is considered in the analysis.

**L.316-317:** ‘Clearly, this group of species does not contribute to the similarity among the ectoparasite communities’ - Species-specific parasites may be more likely to have a phylogenetic signal than generalist parasites (see Hayward et al., 2021). Would it be possible to analyse species specific parasites separate from generalists? You could even compare both results, which would add another interesting point to the study.

- Hayward A, Poulin R, Nakagawa S, (2021). A broadscale analysis of hostsymbiont cophylogeny reveals the drivers of phylogenetic congruence. Ecology Letters. 24:1681–1696. <https://doi.org/10.1111/ele.13757>

A/ We agree with the reviewer, according to the study recently carried out by Osuna-Cabanillas et al 2024 and Hayward et al. 2021, when analyzing specialist and generalist parasites separately, different behaviors are elucidated than in a joint analysis. When performing the analyzes separately in this study, in the analysis of nonspecific parasite species, differences were detected between all host combinations except between *S. gunteri* and *S. papillosum*. For the analysis with only the parasitic species specific to their host, no differences were detected between the hosts, this is perhaps because the sample sizes are too small, and this may cause little ability to detect significant differences between the groups. On the other hand, in both cases significant differences are detected for the region, making it very clear that the Yucatan Peninsula is different from the other two regions.

**L.347-361:** How can the described biogeographical patterns influence fish hosts? Is there any study about the fish hosts distribution, genetic structure, etc? It may be relevant, in particular for specialist parasites, which will only occur where their fish host is available.

A/ Certainly, there is an effect of the biogeographical regions on fish host. In fact, due to this effect, the PERMANOVA in Table 2 shows that host is a significant factor on the ectoparasite species composition.

**Transition Discussion-Conclusion:** The discussion finishes abruptly, similar to the abstract. Overall, the text is very focused on the model system, and the broad context is mostly missing (it is partially mentioned in the conclusion, but since it is missing from the discussion, the conclusion feels like part of the discussion, as it has new information).

A/ Thank you for your comment. We recognize that the discussion lacks a fluid connection to the conclusion. To address this concern, we expand the discussion to provide a more complete framework that places our work within the broader context of research in the field. "Which will help ensure that our results are better contextualized and that the reader can understand their relevance in a broader research context. Additionally, the conclusion was modified.

“In conclusion, the dissimilarity of the structure of the ectoparasitic communities of flatfish in the SGoM seems to be influenced by the presence of specific ectoparasites, which in turn depends on the distribution of the hosts, which may be determined by the topography of the landscape and physical conditions of each region. In contrast, generalist parasites present broader ranges of distribution according to the number of host species that they parasitize. Although, in this study there is no evidence that the phylogenetic affinity between flatfish host species is an essential component in the structure of the ectoparasitic community, it cannot be ruled out. It is possible that this phylogenetic affinity is masked by differences in the local availability of host species that do not allow us to fully estimate how many hosts species are used, or which host are preferred. “It is possible that variation in host availability/abundance by region may be compensated for by analysis on a broader geographic scale and that this will consequently balance interspecific variation among ectoparasitic community structure, which may allow a true phylogenetic signal to be revealed”.

**Minor points - Discussion**

**L.347-353:** This is a single (very long) sentence. Please split it at least into two sentences for clarity.

A/ The sentence was modified as suggested.

**Conclusion**

**L.367-369:** The results of the analyses undertaken do not support ‘phylogenetic affinity’, so based on these results only, it could be ‘ruled out’. However, these analyses do not seem to harness the full potential of the data. There is more that could be done, such as splitting generalist from specialist parasites and accounting for the large differences in sample sizes.

A/ Indeed, for this study the phylogenetic affinity between the hosts based on their ectoparasitic communities was not congruent. However, we do not rule it out because it is likely that the results obtained are masked by the type of sampling. On the other hand, the variability in the size of the samples was considered and this is why we decided to use PERMANOVA diversity analysis with permutations that guarantees the comparison of samples of different sizes. A/ Indeed, for this study the phylogenetic affinity between the hosts based on their ectoparasitic communities was not congruent. However, we do not rule it out because it is likely that the results obtained are masked by the type of sampling. On the other hand, the variability in the size of the samples was considered and this is why we decided to use PERMANOVA diversity analysis with permutations that guarantees the comparison of samples of different sizes. As suggested, analyzes were carried out separating host-specific and non-specific parasitic species. The analysis for nonspecific species detected differences for all host combinations, except S. papillosun and S. gunteri, as did the analysis where all species were included. For the analysis with the host-specific parasitic species, no differences were detected between hosts, this is perhaps because the sample sizes are too small and this may cause little ability to detect significant differences between groups. On the other hand, in both cases significant differences are detected for the region, making it very clear that the Yucatan Peninsula is different from the other two regions.

**Minor points - Conclusion**

**L.370:** Do you mean ‘how many hosts are used’ by a parasite species? Please clarify.

A/ The sentence was modified as suggested.

**Reviewer #3:**

The authors sampled a large number of pleuronectiform fish from 23 different species over a period of 12 years from different regions (grouped into three areas) for ectoparasites. The main aim was to examine the variability on ectoparasite communities in pleuronectiform fish from the area. The authors conclude that variation in these parasite communities are determined primarily by ecology rather than phylogenetics.

The study is well written and does include a large number of samples. However, I do have some concerns that I list below.

For 12 of the 23 species of pleuronectiform fishes sampled, the total sample size is less than 1 per year sampled. As such, given the high variability on prevalence and abundance of parasites in both spatial and temporal spaces, the implications of this unbalanced design need to be discussed.
A/ We appreciate the reviewer's comment. As the reviewer indicates, this study has unbalanced sampling. However, in the diversity analysis using PERMANOVA different sample sizes can be handled by using random permutations. Instead of making direct comparisons between samples with different sizes, random permutations are generated to redistribute the data and create sample groups with similar sizes. This ensures that comparisons are made fairly and that variability in sample sizes is accounted for in the analysis. It should be noted, that for this analysis the most abundant host species that were present in the three regions were used.

I suggest the authors attempt to determine whether their parasite sample for individual host species is representative of the expected ectoparasite fauna. This could be achieved by following the method outlined in Bizzarro JJ, Smith WD, Márquez-FarÌas JF, Tyminski J, Hueter RE (2009) Temporal variation in the artisanal elasmobranch fishery of Sonora, Mexico. Fish Res 97:103–117. If the species accumulation curves do not reach asymptote, this needs to be discussed, i.e. the analyses are based on observed ectoparasite communities, but might not be representative of the “true” ectoparasite diversity of hosts a, b, c, etc.

A/ We understand the reviewer's concern. Unfortunately, this was not a study with directed sampling; On the contrary, all sampling elements had the same probability of being selected with the same sampling effort. Therefore, we followed the reviewer's recommendation and performed a species accumulation analysis to evaluate the sample completeness for each fish species using the sample coverage estimator (Cm), which was included in the manuscript. However, not all species reach asymptote, so, the analyzes were based on observed ectoparasite communities, but might not be representative of the “true” ectoparasite diversity of hosts.

There is no description of the size range sampled for the different pleuronectiform fishes. For instance, due to sampling method (outlined in table 1), is it not possible that authors have a size bias in their sample. Given what is known of the ecology of certain flatfish species, we know that they change substrate and habitat during ontogeny (therefore also salinity and temperature). The length frequencies for each species need to be presented and assessed for signs of size bias. How might this affect the outcome of analyses should then be discussed.

A/ Due to the importance of host size in this study, since these variables affect the parasite composition of flounder species (Alarcos and Timi, 2012; Oliva et al., 2004), we included size of the host as covariate in the new analyses. We found a significant potential effect of host size in parasite composition. However, this effect seemed to be constant among regions and hosts since there were not significant interactions with the other factors (Tabla 2). Additionally, we include the length range (LT) by species in table 2.

It is not clear whether host identified only to genus level could be conspecific with others sampled in this study. This needs to be outlined clearly.
A/ As it is no longer possible to verify whether the hosts identified at the genus level are conspecific with the sampled species, we decided to eliminate individuals not identified to species from this study (see Table 1).

For the phylogenetic relationships within the order, authors added 32 species of the same families. However, there is no table listing these species and their taxonomic affinities. This is shown via a figure, but without GenBank accession numbers and taxonomic affinities provided, it is difficult to assess the adequacy of included taxa. Additionally, there is no rationale provided as to why these 32 taxa were selected. Given the apparent bias in taxa selected for phylogenetic analyses, it is possible that the trees are not similar with the “true” topology due to gaps in taxonomic sampling for phylogenetic analyses. Was the topology compared to that of a tree for the Pleuronectiformes (or similar)?

A/ We do not believe a table is necessary in the main document because in the phylogenetic tree, the Genbank accession number of the sequence used is placed in front of the name of each species, and the classification of each species at the family level is indicated with the color of each branch of the tree. The color/family relationship is indicated in the figure legend.

The species of flatfishes considered in this study were selected because: 1) the species most closely related to the species reviewed in this study, that had published cox 1 sequences in Genbank, which were distributed in the North Atlantic and in the Gulf of Mexico; and 2) in comparison with a Pleuronectiformes tree (Campbell et al., 2019) that allowed us to know the phylogenetic position of the families, representatives of the families to which the reviewed hosts belong were selected.

There seems to be an error in reporting prevalence in the Abstract and Results with prevalence ranging from 0.01 to 37.5%. Since fewer than 2000 hosts were sampled, I would expect the prevalence to range from 0.1 to 37.5%. Check other calculations as well.

A/ The reviewer is correct, there is a typographical error in the prevalence range, which was already corrected in the Abstract and Results.

“The overall prevalence ranged between 0.1% for *Argulus* sp., *Gnathia* sp. and *Trachellobdella lubrica* from *Cyclopsetta chittendeni* and 37.5% for *Bomolochus* sp1 from *Trinectes maculatus*”

Furthermore, there is likely a “year” effect that should be assessed. Since the study spans 12 years and we know that parasite communities fluctuate in space and time, it is not unreasonable to suggest that year of sampling could affect the results.

A/ As suggested, years effect was included in the analyzes because it is considered an important factor in the structure of the ectoparasitic community. For this, we additionally performed an analysis of similarities (ANOSIM) for the sampling dates. However, no relevant year effect was found that affects the structure of the ectoparasitic community of the different species of flatfish collected in different cruises (see the following table, included in the manuscript as S3 Table).

**Table** ANOSIM analysis results for the sampling dates**.**

|  | R Significance | |
| --- | --- | --- |
| Groups | Statistic | *P* value |
| 2008, 2016 | -0.333 | 1 |
| 2008, 2017 | 1 | 0.333 |
| 2008, 2018 | 0.583 | 0.220 |
| 2010, 2016 | -0.556 | 1 |
| 2010, 2017 | 0 | 0.667 |
| 2010, 2018 | -0.5 | 100 |
| 2011, 2016 | -0.556 | 100 |
| 2011, 2017 | 0 | 0.667 |
| 2011, 2018 | -0.333 | 0.80 |
| 2012, 2016 | -0.333 | 1 |
| 2012, 2017 | 0 | 0.667 |
| 2012, 2018 | -0.375 | 1 |
| 2015, 2016 | -0.556 | 1 |
| 2015, 2017 | 0 | 0.667 |
| 2015, 2018 | 0.417 | 0.40 |
| 2016, 2017 | -0.417 | 1 |
| 2016, 2018 | 0.009 | 0.429 |
| 2017, 2018 | -0.25 | 0.80 |

I don’t think that the conclusions reached match the data. From the data, as analysed, it would seem that there is no phylogenetic signal. However, there was no assessment of environmental variables in this study, therefore, authors cannot conclude that the variability on ectoparasite communities is due to ecology. I have provided some comments on other uncertainties that could cloud the outcome. Without a proper analysis of effects on specific environmental variables on ectoparasite communities, authors cannot conclude that the variation is due to the environment.

A/ Certainly, there was no assessment of environmental variables in this study, basically because these data were incomplete. However, the emphasis of our study was to determine what was the proportion of ectoparasites species belonging to the specialist group (what we called the phylogenetic component) and the generalist group (what we called the ecological component) across the southern Gulf of Mexico. We called this group of ectoparasites the ecological component basically because they can be acquired by the flatfishes from the regional pool of parasites available in the southern Gulf of Mexico independently of their co-evolutionary history with the host. In contrast, the phylogenetic component of the parasite fauna are those ectoparasites with a large co-evolutionary history with the host. The results showed that the proportion of species belonging to the ecological component is much higher than that of the phylogenetic component. This means that the explanations on the availability of these ectoparasites to flatfishes are related more to the environmental condition prevailing in each of the three geographical regions compared. Now, based on the literature referring to the regionalization of the Gulf of Mexico (Zavala-Hidalgo et al. 2003, Salmeron-Garcia et al. 2011, Quintanilla-Mena et al. 2020), we can at least infer that relevant environmental factors that could be affecting the availability of ectoparasite species of this ecological component would be meteorological processes, physical oceanographic processes, river discharges and topographic characteristics of the ocean.

Looking forward to hearing from you soon

Yours sincerely

___________________________

Lilia Catherinne Soler-Jiménez

Aquatic Pathology Laboratory

CINVESTAV-IPN Mérida

**References**

- Anderson M, Gorley RN, Clarke KR (2008) PERMANOVA + for PRIMER: Guide to software and satistical methods (Vol. 1). PRIMER-E Ltd.
- Alarco AJ, Timi JT (2012) Parasite communities in three sympatric flounder species (Pleuronectiformes: Paralichthyidae). Parasitol Res 110:2155–2166. doi:10.1007/s00436-011-2741-5.
- Atta et al., 2022
- Bush AO, Lafferty KD, Lotz JM, Shostak AW (1997) Parasitology meets ecology on its own terms: Margolis et al. revisited. J Parasitol 83:575–583. doi:10.2307/3284227.
- Campbell MA, Chanet B, Chen JN, Lee MY, Chen WJ (2019) Origins and relationships of the Pleuronectoidei: Molecular and morphological analysis of living and fossil taxa. Zool Scr 48(5):640–656.
- Hayward A, Poulin R, Nakagawa S (2021). A broadscale analysis of hostsymbiont cophylogeny reveals the drivers of phylogenetic congruence. Ecology Letters 24:1681–1696. https://doi.org/10.1111/ele.13757
- Oliva ME, González MT, Acuña E (2004) Metazoan parasite fauna as a biological tag for the habitat of the flounder *Hippoglossina macrops* from Northern Chile, in a depth gradient. J Parasitol 90(6):1374–1377. https://doi.org/10.1645/GE-317R
- Osuna-Cabanillas et al 2024
- Quintanilla-Mena et al [2020]
- Salmerón-García O, Zavala-Hidalgo J, Mateos-Jasso A, Romero-Centeno R (2011) Regionalization of the Gulf of Mexico from space-time chlorophyll-a concentration variability. Ocean Dyn 61(4):439–448. doi:10.1007/s10236-010-0368-1.
- Zavala-Hidalgo J, Morey SL, O’Brien JJ (2003) Seasonal circulation on the western shelf of the Gulf of Mexico using a high-resolution numerical model. J Geophys Res 108(C12):3389. doi:10.1029/2003jc001879.
