# Supplementary material for: Ecological and phylogenetic components of flatfish ectoparasites (Pleuronectiformes: Paralichthyidae) from the Southern Gulf of Mexico
Source: PLoS One. 2024 Oct 24;19(10):e0309818. doi: 10.1371/journal.pone.0309818 (PMC11500868; doi:10.1371/journal.pone.0309818)
Supplement: Attachment — Submitted filename: Response to Reviewers R1.docx [file pone.0309818.s007.docx]

Lilia C. Soler-Jiménez

Aquatic Pathology Laboratory

CINVESTAV

June 27, 2024

Professor **Joshua Kamani**, PhD

Academic Editor

PLOS ONE

Dear Dr. Joshua Kamani

Attached you will find the manuscript entitled **“Ecological and phylogenetic components of flatfish ectoparasites (Pleuronectiformes: Paralichthyidae) from the Southern Gulf of Mexico”** by Lilia C. Soler-Jiménez, Frank A. Ocaña, David I. Hernández-Mena, Oscar A. Centeno-Chalé, Ma. Leopoldina Aguirre-Macedo and Víctor M. Vidal-Martínez. All the editorial suggestions were considered in this new version of the manuscript. Below you will find detailed answers to each one of these suggestions.

**AUTHOR(S):** Lilia C. Soler-Jiménez, Frank A. Ocaña, David I. Hernández-Mena, Oscar A. Centeno-Chalé, Ma. Leopoldina Aguirre-Macedo and Víctor M. Vidal-Martínez.

**Manuscript number:** PONE-D-23-31417R1

**TITLE:** Ecological and phylogenetic components of flatfish ectoparasites (Pleuronectiformes: Paralichthyidae) from the Southern Gulf of Mexico.

We are truly grateful with the referees for their valuable comments. We included first the referee´s suggestion and/or question, followed by our answer in a paragraph starting with A/, an acronym that stands for “Answer”, and in blue color.

**Response to Reviewers**

Reviewer #1: The authors have resolved many of the issues raised in the first revision, resulting in a much more attractive manuscript. However, some issues still need to be resolved.

In lines 66 to 70 and lines 75 to 77, the authors aim to assess the contribution of ecological components to the similarity of flatfish ectoparasite communities across the SGoM. It can be seen in the data analysis section that the depths of the regions investigated (line 176), as well as the geographical distances between them (lines 189 and 190) were considered in the analyses. Do these variables correspond to the only ecological components considered? If so, this should be clearly defined in the objectives.

Why was the similarity of host species between regions not considered as an explanatory variable?

A/ The reviewer is correct, depth and geographical distance were the only ecological components included in the statistical analyses. However, these were not the only components considered in the overall analysis of the similarity of flatfish ectoparasite structure in the SGoM. Therefore, we do not consider it appropriate to modify the objectives.

Similarity of host species between regions was not considered as an explanatory factor in our study to maintain focus and consistency with the original objectives of the study. We focused on assessing the relative contribution of phylogenetic and ecological components to the similarity of flatfish ectoparasite communities throughout the study area.

**Minor reviews:**

line 167: typo error "e.i." for "i.e.";

A/ Typographical error corrected. We thank the reviewer for his comments.

lines 167 to 169: insert in the text the reason for transforming the raw data;

A/ The reason for transforming the raw data was included.

“Ectoparasite abundance data of selected hosts (i.e. species of parasitized hosts present in all regions and with more than five individuals in each region) were transformed square roots to reduce the contribution of highly abundant species in relation to less abundant ones”

line 203: typo error "Gulf of Mexico. , and which"

A/ Typographical error corrected.

Reviewer #2: The authors of the manuscript entitled “Ecological and phylogenetic components of flatfish ectoparasites (Pleuronectiformes: Paralichthyidae) from the Southern Gulf of Mexico” dedicated great effort to this revised version of the manuscript, and to my view the new version is a significant improvement from the original submission. Relevant analyses were added and the introduction and discussion now clearly identify the broader context in which this work is placed. My main concerns are now (1) a number of grammatical errors and some poor sentences that should be corrected and improved before publication, (2) that there still seems to be some emphasis in understanding co-evolution between hosts and parasites when the authors said in the response letter that their data lacks the resolution for a formal test of the relationship between host and parasite phylogenies. I made a few suggestions below to ‘tone down’ the co-phylogeny emphasis, as well as some suggestions for grammar/sentence structure (although I did not make an exhaustive search of all the errors, so I recommend the authors proof-read their manuscript prior to publication).

A/ We thank the reviewer for his suggestions to improve understanding of the coevolution between hosts and parasites. In addition, the manuscript was thoroughly reviewed for grammatical or structural errors.

I should also mention I appreciate that the authors now analysed ecological components using depth and host size. Their results were significant, so I think at least a sentence about it should be in the discussion. In L 351, does “basic ecological processes” mean depth, size, and host geographical structure? If so, these three factors could be simply named there.

A/ We appreciate the reviewer's comment. As suggested, we mentioned the ecological components analyzed in the discussion.

Finally, regarding PERMANOVA and sample sizes, the reason why I mentioned sample sizes and rarefaction is not because of the statistical analysis used, but because of the probability of finding parasites. If a parasite has a 1% prevalence and you collect 5 fish of species A but 100 fish of species B, you will most likely find it only on species B. However, the authors did include a rarefaction analysis in their new version and clearly explained that the diversity in most cases did not level off, which in my opinion is all that is needed. Overall, I think the authors carefully considered all the comments made by reviewers and addressed all of the suggestions either by modifying the manuscript or by explaining their rationale in the response letter. The new version is even more interesting than the previous one, and much more grounded in the available data and analyses undertaken. All my comments below refer to the revised manuscript without track-changes.

A/ We appreciate the reviewer´s comment and suggestions to improve the manuscript.

**Specific comments about the emphasis on co-evolution**

In the response letter, the authors stated that “the objective of the analysis was only to demonstrate that the reviewed flatfish do not have a close phylogenetic relationship.” In lines 199-200, the authors wrote "… to test the hypothesis that a phylogenetic component of their hosts may influence ectoparasite community structure". I would replace it by something like "… to test how closely related the flatfish species are, so that hosts phylogeny can be considered as a factor explaining ectoparasite community structure". The current sentence “to test the hypothesis that a phylogenetic component of their hosts may influence ectoparasite community structure”, gives the impression that the authors are testing for co-phylogeny, but the authors themselves said in the response letter “… that the objective of our analyses was to investigate whether or not the assembly of parasite communities has a relationship with the phylogeny of the hosts, rather than the coevolution between parasites and specific hosts”, and that the fish phylogenetic trees lack resolution for them to make phylogenetic inferences. So, in my opinion the only modification needed is to downplay a little the “coevolution hypothesis” to match exactly what was explained in the response letter.

A/ Thank you for your comments. As you point out, the goal of our analyzes was to investigate whether or not the assembly of parasite communities has a relationship with host phylogeny, rather than coevolution between parasites and specific hosts. Therefore, downplaying the coevolution hypothesis is appropriate, as you suggested.

For example, in L211-214, it reads “Subsequently, to check if the ectoparasite community structure of each host species had any congruence with the phylogenetic relationships of the fish, two analyses were carried out to group the fish based on the parasites species shared: one of similarity and another of parsimony.” I am not sure if “phylogenetic congruence” is the best wording here, maybe “to check if ectoparasite communities are more similar in closely related hosts than in hosts more phylogenetically distant”?

A/ The phrase was modified to clarify the idea, as suggested:

“to test how closely related the flatfish species are, so that hosts phylogeny can be considered as a factor explaining ectoparasite community structure”

I also think the authors highlighted a valuable point in the response letter that is not mentioned in the text, and I quote “However, for us it was important that all fish species reviewed in this study were represented in the phylogeny, and the only gene that has been sequenced for all of them is cox 1.” I think a more polished version of this sentence could be added to the text, maybe in the methods or discussion. In addition to supporting the analyses in the way the authors have undertaken them (only with cox1), this sentence highlights a major gap in fish genetics.

A/ We appreciate the reviewer's comment. As suggested, the reason for using the Cox1 gene expressed in the response letter was also included in the Materials and Methods section of the manuscript:

“It should be noted that for this study it was important that all fish species included in this study were represented in the phylogenetic analysis, and the only gene that has been sequenced for all of them is cox 1”

Specific comments about grammar/sentence structure

L27 Remove comma before ‘presumably’ in “… in those species that, presumably”

A/ Typographical error corrected as suggested.

L52 “Neady localities” – do you mean nearby?

A/ Typographical error corrected as suggested.

L51-53 Please re-write this sentence to make it less repetitive “Thus, we would expect that in nearby localities, the similarity of the parasite communities in those localities would be high in neardy localities, but with increase in the geographical distance, it would decrease.”

One suggestion is “Thus, we would expect that in nearby localities, the similarity of the parasite communities would be high, decreasing with increased geographical distance”

A/ We appreciate the reviewer´s comment. The phrase was rewrite as suggested:

“Thus, it would be expected that in nearby locations, the similarity of parasite communities would be high, decreasing with increased geographical distance”.

L53 “Increase in the geographical distance” – replace by ‘increased geographical distance’

A/ The phrase was modified as suggested.

L.53 Remove “What is”

A/ “What is” was removed from the phrase as suggested.

L182-183 “Additionally, an analysis of similarities (ANOSIM) was performed for the sampling dates.” – Add the reason why this was done to make it clearer for the reader. I suggest continuing the sentence with “to check for potential effects on parasite communities of sampling across different years.”

A/ The idea was completed as suggested:

“Additionally, an analysis of similarities (ANOSIM) was performed for the sampling dates to check for potential effects on parasite communities of sampling across different years”.

L203 Remove full stop after ‘Gulf of Mexico’

A/ The full stop was removed as suggested.

L246-248 Check grammar: ‘but not be representative’ should be ‘but are not representative’

A/ Typographical error corrected as suggested.

L257-258 Check grammar and clarity in the following sentence: “However this effect seemed constant among regions and hosts since there were not significant intereactions were found between these factors”.

- Interactions instead of interactions

- Correct “there were not significant interactions were found”

- It is not clear what “this effect” and “these factors” refer to, please write in full instead of using “this” and “these”

The sentence the authors wrote in the response letter is actually much clearer, and could be used instead: “We found a significant potential effect of depth in parasite composition. However, this effect seemed to be constant among regions and hosts since there were not significant interactions with the other factors.”

A/ We rewrite the sentence to clarify the idea, as suggested:

“However, this effect seemed to be constant among regions and hosts since there were not significant interactions between host size and depth or with the other factors”

L260 “… but no significant interaction was detected between regions and hosts”. This statement seems repetitive with the one in lines above (L257-258), but I can’t be sure because the statement in L 257-258 is confusing. Consider removing the repetition from L260 if redundant.

A/ The statement was rewritten to avoid repetition and confusion, as suggested:

“A significant potential effect of host size and depth in parasite species composition was detected. However, this effect seemed to be constant among regions and hosts since there were not significant interactions between host size and depth or with the other factors”

L335 (legend of Fig3): Remove bold font from the P in ‘Parsimony’

A/ Typographical error corrected as suggested.

L360 “…parasite species give…” do you mean “are responsible for” or “are driving the results of similarity” instead of “give”?

A/ The phrase was modified as suggested:

“However, it must be considered that the nonspecific parasite species are responsible for the similarities between the ectoparasitic structure in these host species”

L363 Instead of “species parasites” use “parasite species”

A/ The error was corrected as suggested.

L428, 438, and 441 – Remove quotation marks from Conclusion

A/ Typographical error corrected as suggested.

L712 Please translate the table legend to English

A/ The error was corrected as suggested.

Looking forward to hearing from you soon

Yours sincerely

___________________________

Lilia Catherinne Soler-Jiménez

Aquatic Pathology Laboratory

CINVESTAV-IPN Mérida
